# Nanoporous Gold Nanoparticles-Modified Electrode for the Detection of Endotoxins

**DOI:** 10.3390/mi16091014

**Published:** 2025-08-31

**Authors:** Dhanbir Lingden, Preston Willis, Jay K. Bhattarai, Keith J. Stine

**Affiliations:** 1 Department of Chemistry and Biochemistry, University of Missouri–Saint Louis, Saint Louis, MO 63121, USA; dlv4f@umsl.edu (D.L.); jkbxv3@mail.umsl.edu (J.K.B.); 2 Department of Chemistry, College of Arts and Sciences, Case Western Reserve University, Cleveland, OH 44106, USA; pmw54@case.edu

**Keywords:** nanoporous gold nanoparticles (np-AuNPs), biosensing, lipopolysaccharide (LPS), endotoxin, sepsis, inflammation

## Abstract

Nanoporous gold nanoparticles (np-AuNPs) combine inertness, a nanoscale structure, and a porous framework with high surface area, conductivity, and biocompatibility, making them ideal for biosensing, catalysis, fuel cells, and drug delivery. Their open pore structure and low-coordinated atoms enhance biomolecule capture and mass transfer, while their tunable size, pore volume, and ease of surface modification make them promising biosensor transducers. However, synthesizing colloidal np-AuNPs in a simple way with controllable size and scalability remains challenging. The existing approaches mostly rely on specialized equipment, complex setups, and expert knowledge, while still facing challenges in terms of scalability. In this study, we present a simple, seedless, wet-chemical synthesis of colloidal np-AuNPs via the co-reduction of Au/Ag alloys followed by dealloying. By adjusting the Au:Ag ratio, we produced np-AuNPs sized ~120–530 nm, which were immobilized on electrodes for detecting lipopolysaccharide (LPS), a toxic component of Gram-negative bacterial membranes. The LPS biosensor exhibited excellent sensitivity towards detecting wild-type LPS, with a low limit of detection (LOD) of 0.1244 ng/L. This work demonstrates the effective synthesis and application of np-AuNPs in LPS biosensing.

## 1. Introduction

Lipopolysaccharide (LPS) is a key contributor to sepsis and septic shock, a severe, systemic inflammatory response to bacterial infection that results in millions of deaths worldwide [1]. Even at concentrations as low as 1 ng/mL, LPS can activate the immune system and trigger inflammation [2]. Approximately 1% of all hospital patients, and about 20–30% of those in intensive care units (ICUs), are estimated to develop sepsis [3]. According to the Global Burden of Disease Study 2017, there were 48.9 million cases of sepsis globally that year, with 11 million resulting in death [4]. A meta-analysis by Fleischmann-Struzek et al. further estimated a mortality rate of 26.7% among sepsis patients, with a 46% rise in incidence since 2008 [5]. The global incidence rate stands at roughly 677.5 cases per 100,000 people [4]. In North America, the rate ranges from 500 to 1000 cases per 100,000, with the United States reporting higher numbers than Canada [6,7]. The highest rates are observed in several low- and middle-income countries in Africa and South Asia, where sepsis affects more than 1500 individuals per 100,000 [8,9,10].

Given the severity and widespread impact of sepsis, there is an urgent need for sensitive and selective methods for LPS detection. While traditional techniques are still in use, modern biosensors, particularly those based on nanomaterials, are emerging as promising tools due to their high sensitivity and rapid response times. Among these, nanoporous gold nanoparticles (np-AuNPs) are especially notable for their stability, biocompatibility, and ease of surface modification [11,12].

Nanoporous gold nanoparticles (np-AuNPs) are a unique combination of a stable, inert material, a nano-sized structure, and a porous framework, featuring nanoscale pores and ligaments. Due to their high surface area, excellent conductivity, and biocompatibility, np-AuNPs are highly suitable for applications in biosensing, catalysis, fuel cells, and drug delivery [12,13]. Additionally, they are distinguished by their enhanced ability to capture biomolecules and improve the mass transfer of reactants through their highly active low-coordinated atoms and open pore structures. Their tunable size and pore volume, along with the ease of surface modification, further underscore their potential for biosensor development. These characteristics make np-AuNPs a promising material for use as transducers in biosensing. However, the simple and effective synthesis of np-AuNPs in colloidal form with controllable size remains a challenge.

Although the synthesis of nanoporous gold structures has been explored for some time, significant advancements in the production of nanoporous gold nanoparticles (np-AuNPs) seem to have begun around 2012, when D. Wong and P. Schaaf first reported the synthesis of np-AuNPs via a dewetting method followed by a dealloying process [14]. Since then, numerous studies have been conducted in this field; however, many of the methods for synthesizing np-AuNPs still lack simplicity and efficiency. One of the most commonly used techniques, dewetting followed by dealloying [14,15,16,17,18,19,20,21,22,23,24,25,26,27,28,29], requires high temperatures and extended processing times. Other approaches, such as seed-mediated methods [30,31,32,33,34,35], involve sacrificial templates to create nanopores within gold nanoparticles, while electrochemical methods [36,37,38,39] are time-intensive and require specialized expertise due to their complex setups. Considering these challenges, a simpler and more efficient synthesis method for np-AuNPs would be ideal. In this study, we introduce a straightforward and effective solution-phase synthesis of colloidal np-AuNPs using a wet-chemical process. This method involves the seedless synthesis of gold/silver (Au/Ag) alloy nanoparticles through co-reduction, followed by chemical dealloying. The resulting np-AuNPs are then immobilized onto an indium tin oxide (ITO)-coated glass electrode to create a biosensing platform for the sensitive detection of LPS.

## 2. Materials and Methods

### 2.1. Chemicals

Gold (III) chloride trihydrate (HAuCl_4_·3H_2_O) (≥99.9%, trace metals basis), silver nitrate (AgNO_3_) (≥99%, ACS reagent), L-ascorbic acid (C_6_H_8_O_6_, BioXtra, ≥99.0%, crystalline), polyvinylpyrrolidone (PVP; average mol wt. = 40,000), ethanol (≥99.5%), (3-aminopropyl)triethoxysilane (APTES, 99%), N-(3-dimethylaminopropyl)-N′-ethylcarbodiimide hydrochloride (EDC, ≥99.0%), N-hydroxysuccinimide (NHS, ≥97.0%), (±)-∝-Lipoic acid (≥99%), bovine serum albumin (BSA), sodium chloride (99.999%), potassium chloride (99.999%), Potassium hexacyanoferrate(III) (K_3_ [Fe(CN)_6_]) (≥99.99%), potassium hexacyanoferrate(II) trihydrate (K_4_ [Fe(CN)_6_]·3H_2_O) (≥99.99%), Sodium phosphate dibasic (Na_2_HPO_4_) (≥99.5%), Trizma^®^ base (≥99.9%), calcium chloride dihydrate (CaCl_2_·2H_2_O) (≥99.5%), manganese(II) chloride tetrahydrate (MnCl_2_·4H_2_O) (≥99.99%), and lipopolysaccharides (LPSs) from *Escherichia coli* O55:B5, were purchased from Sigma-Aldrich, St. Louis, MO, USA (currently known as Millipore Sigma, St. Louis, MO, USA). Lipid A LPS polyclonal antibody (4–5 mg/mL) was bought from Invitrogen (a brand owned by Thermo Fisher Scientific, Waltham, MA, USA). Concanavalin A (Con A, unconjugated) was purchased from Vector Laboratories (Newark, CA, USA). Nitric acid (HNO_3_, certified ACS plus), ammonium hydroxide (certified ACS Plus), and hydrochloric acid (HCl, OPTIMA^TM^) were obtained from Thermo Fisher Scientific. Potassium phosphate monobasic (KH_2_PO_4_) (≥99.5%) was purchased from Fluka, BioChemika (now part of Honeywell Research Chemicals, Charlotte, NC, USA). All chemicals were used without further purification. Milli-Q water (>18 MΩ cm) was obtained using Milli-Q IQ 7010 system (Millipore Sigma, St. Louis, MO, USA).

### 2.2. Synthesis of Nanoporous Gold Nanoparticles (np-AuNPs)

**Figure 1:**
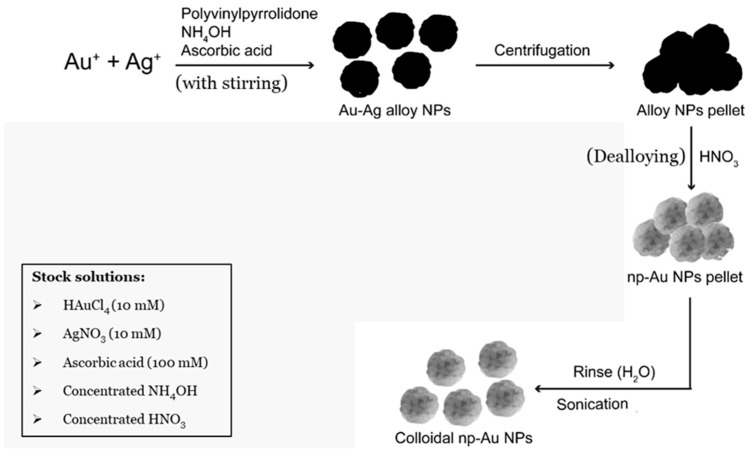
Schematic representation of synthetic scheme of nanoporous gold nanoparticles (np-AuNPs).

We first started by aiming to make a final volume of 10 mL. First, 0.2 g of PVP (2% of PVP (weight of PVP in grams/total volume in mL)) for the 10 mL final volume (to make a final PVP concentration of 0.5 mM), was dissolved in 9 mL of Milli-Q water in a 20 mL scintillation vial with continuous stirring using aqua regia-cleaned magnets. After the complete dissolution of the PVP, 50 μL of concentrated NH_4_OH (0.5% *v*/*v*) was added to the vial. Afterwards, 125 μL of 10 mM gold (III) chloride and 375 μL of 10 mM silver nitrate solution (total volume of gold (III) chloride and silver nitrate was 5% (*v*/*v*)) were added to the vial simultaneously in order to make a Au:Ag molar ratio of 1:3. Immediately following this, 450 μL of 100 mM L-ascorbic acid (4.5% *v*/*v*) was added to the solution. A change in the color of the solution from colorless to reddish brown indicated the formation of gold/silver (Au/Ag) alloy nanoparticles, which was later confirmed by scanning electron microscopy (SEM). The Au/Ag alloy nanoparticles were then washed with Milli-Q water using repeated centrifugations at 5000 r.p.m. for 5 min. Finally, the alloy nanoparticles were dealloyed using concentrated nitric acid to form nanoporous gold nanoparticles (np-AuNPs), washed multiples times with Milli-Q water, redispersed in Milli-Q water using sonication, and stored at 4–8 °C for long-term stability. The final volume was scaled up and no issues were observed up to a volume of 1 L. A general representation of this method is illustrated in Figure 1.

### 2.3. Scanning Electron Microscopy (SEM) Analysis

Scanning electron microscopy (SEM) analyses were conducted using an Apreo 2 High Vacuum FEG SEM (Thermo Fisher Scientific, Waltham, MA, USA). The SEM was employed to characterize the nanoporous gold nanoparticles (np-AuNPs) as well as the modified electrode surfaces. The imaging was performed at working distances ranging from 5 mm to 10 mm, with an accelerating voltage between 5 kV and 20 kV, and beam currents varying from 50 pA to 0.4 nA. The elemental composition was determined by energy-dispersive X-ray spectroscopy (EDS) through ChemiSEM technology incorporated into the SEM. For the EDS measurements, a constant working distance of 10 mm was used with the accelerating voltage set between 10 kV and 20 kV and the beam currents ranging from 0.1 nA to 1.6 nA. The size measurements were performed using ImageJ software (version 1.54d), a Java-based image processing software developed at the National Institutes of Health (NIH), Bethesda, MA, USA.

### 2.4. Transmission Electron Microscopy (TEM) Analysis

Transmission electron microscopy (TEM) and scanning transmission electron microscopy (STEM) were also used to confirm the formation of these nanoporous gold nanoparticles (np-AuNPs). The TEM and STEM high-angle annular dark-field (HAADF) imaging were conducted on a Thermo Fisher Spectra 300 instrument operated at 300 kV. The STEM-EDS elemental mapping was acquired on the Spectra 300 with Super-X EDS technology. The high-resolution TEM images were obtained using Ceta-camera of TEM.

### 2.5. Dynamic Light Scattering (DLS) Analysis

The dynamic light scattering (DLS) analysis was performed with a DynaPro Titan Instrument (Wyatt Technology, Santa Barbara, CA, USA) to observe the size distribution of these np-AuNPs prepared using a Au-to-Ag molar ratio of 1:3. A total of 35 μL of sample was placed into a quartz cuvette, and the light scattering intensity was collected at a 90° angle using an acquisition time of 5 s. The mean diameter was calculated from the mean hydrodynamic radius of the particles, which was obtained using Dynamics software (version 6.12.03).

### 2.6. UV-Vis Spectroscopy Analysis

UV-Vis and LSPR spectra were used to the characterize spectral properties of the Au/Ag alloy nanoparticles and nanoporous gold nanoparticles. It was performed using a Cary 50 UV–Visible spectrophotometer (Varian Inc., Palo Alto, CA, USA, which was acquired by Agilent Technologies in 2010).

### 2.7. Modification of Electrode with np-AuNPs

**Figure 2:**
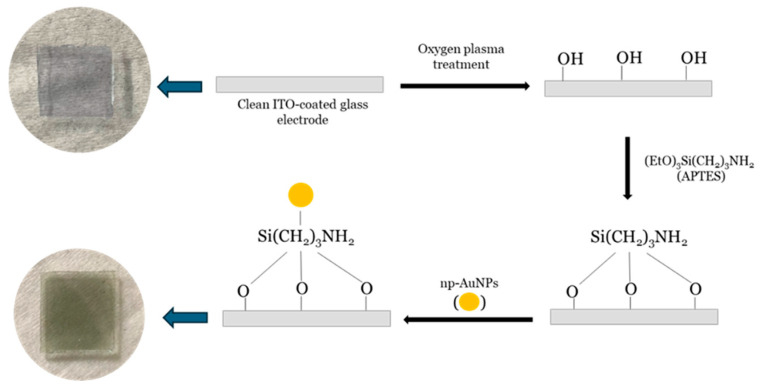
Schematic illustration of method used for modification of ITO-coated glass electrode surface with np-AuNPs. The large arrows point left towards photographs of the ITO glass piece before and after the steps indicated by the black arrows.

The working electrode was prepared by modifying an indium tin oxide (ITO)-coated glass electrode with nanoporous gold nanoparticles (np-AuNPs). The ITO-coated glass was first cut into approximately 1 × 1 cm^2^ pieces and thoroughly cleaned using Milli-Q water and ethanol, each with sonication repeated at least three times. After cleaning, the electrodes were dried with nitrogen (N_2_) gas and then treated with oxygen (O_2_) plasma for a minimum of 15 min to enhance the surface oxygen species, such as hydroxyl (-OH) groups, and to remove organic contaminants. The electrodes were subsequently immersed in a 1% (*v*/*v*) APTES solution in ethanol for at least one hour. Following this, they were rinsed with ethanol and Milli-Q water to eliminate any loosely bound materials and dried again under a nitrogen flow. Finally, np-AuNPs were drop-casted onto the APTES-functionalized ITO-coated electrodes and left overnight in a covered fume hood to allow for immobilization. This process yielded np-AuNPs-modified ITO-coated glass electrodes. The process is illustrated in Figure 2.

### 2.8. Modification of Electrode for Biosensing LPS

As mentioned in the previous section, the surface of clean ITO-coated glass electrodes was modified with np-AuNPs using an O_2_-plasma treatment followed by APTES functionalization. The O_2_-plasma treatment helped remove organic contaminants from the electrode surface, making the hydroxide bonds more available for APTES [40,41]. APTES is a well-known linker used to immobilize gold nanoparticles through electrostatic interactions between its amine groups and gold nanoparticles [42,43,44]. The np-AuNPs-modified electrodes were then treated with 10 mM lipoic acid (in ethanol) overnight and rinsed with ethanol and acetonitrile at least three times each. This was followed by EDC-NHS chemistry to activate carboxylic groups of lipoic acid to attach a lipid A of LPS-specific antibody. Lipoic acid is widely used in various applications, including biosensing, due to its ability to interact with various biological molecules [45,46]. The lipoic acid-treated electrodes were then immersed in a solution containing a mixture of 5mM EDC and 5 mM NHS for at least 5 h. Then, the electrodes were rinsed with acetonitrile at least three times, dried using N_2_ gas, and again rinsed with 10 mM PBS buffer (pH 7.2). After this step, the modified electrodes were left immersed overnight in a 10 mL solution containing 80–100 μg of lipid A LPS polyclonal antibody. The lipid A LPS polyclonal antibody was used for binding lipid A segment of LPS molecules with specificity and accuracy. The antibody-functionalized electrodes were then treated with a blocking buffer, 1% BSA in 10 mM PBS buffer (pH 7.2) (i.e., 1 g BSA in 100 mL of buffer), for at least 30 min to prevent non-specific binding of the antibody by competing for the binding sites, thus reducing the background noises and improving the signal-to-noise ratios [47,48]. The modified electrodes were then rinsed with 10 mM PBS (pH 7.2) and stored at 4–8 °C in 10 mM PBS buffer (pH 7.2) for further use as the biosensing platforms.

### 2.9. Cyclic Voltammetry (CV)

Cyclic voltammetry (CV) was employed to analyze the surface properties of ITO-coated glass electrodes modified with nanoporous gold nanoparticles (np-AuNPs). The measurements were conducted using a PARSTAT 2273 potentiostat/galvanostat (Princeton Applied Research, Oak Ridge, TN, USA; currently under AMETEK Scientific Instruments). A three-electrode configuration was utilized, comprising the modified electrode as the working electrode, an Ag/AgCl reference electrode, and a platinum wire counter electrode. The redox couple Fe(CN)_6_^4−^/^3−^ at pH 7.2 served as the electrochemical probe. The CV scans were carried out over a potential range from −0.2 V to +0.6 V at a scan rate of 0.1 V/s and a pulse amplitude of 5 mV.

### 2.10. Electrochemical Impedance Spectroscopy (EIS)

EIS was employed to evaluate the charge transfer resistance (R_ct_), overall impedance (Z), and phase angle (φ) of the modified electrodes for their characterization and biosensing. The measurements were carried out using a PARSTAT 2273 potentiostat/galvanostat (Princeton Applied Research, Oak Ridge, TN, USA; now part of AMETEK Scientific Instruments). A redox probe consisting of 5 mM K_3_ [Fe(CN)_6_]/K_4_ [Fe(CN)_6_] in a 1:1 ratio was prepared in 10 mM phosphate buffer at pH 7.2 with 100mM KCl as the supporting electrolyte. EIS was performed over a frequency range from 100 kHz to 0.1 Hz, using a bias potential of 0.2 V (vs. the reference electrode), with a sampling rate of 12 points per decade and an AC amplitude of 10 mV. The data were analyzed using ZSimpWin 3.21 software (Princeton Applied Research, Oak Ridge, TN, USA).

## 3. Results and Discussion

### 3.1. Synthesis of np-AuNPs

We have introduced a straightforward method for synthesizing colloidal np-AuNPs in solution using a wet-chemical approach. As noted earlier, the synthesis process includes the seedless preparation of gold/silver (Au/Ag) alloy nanoparticles through co-reduction with ascorbic acid, followed by dealloying using nitric acid. Ascorbic acid is commonly used as a reducing agent in the synthesis of gold nanoparticles. During this process it reduces Au(III) and Ag(I) ions to their metallic forms by donating electrons, leading to the formation of Au/Ag alloy nanoparticles [49,50]. Polyvinylpyrrolidone (PVP) serves to stabilize the np-AuNPs in solution. It functions as both a capping and stabilizing agent during synthesis by binding to the surface of the nanoparticles, preventing aggregation and encouraging the formation of smaller, more uniform particles. This stabilization is mainly achieved through steric hindrance, where the PVP chains physically keep the nanoparticles from coming together and clumping. Additionally, the lactam ring in PVP can interact with the surface of the np-AuNPs, further aiding in stabilization [51,52]. Ammonium hydroxide is used to facilitate the dissolution of AgCl by forming a soluble complex with Ag(I) ions through the donation of electron pairs [53]. Since AgCl is only partially soluble in water, it could impede the formation of np-AuNPs. Finally, nitric acid selectively dissolves the less noble metal in the alloy, silver, by converting it into AgNO_3_, allowing for the formation of np-AuNPs in solution.

### 3.2. SEM/EDS Characterization

SEM/EDS was used to analyze the formation of Au/Ag alloy nanoparticles and nanoporous gold nanoparticles (np-AuNPs). For preparing the samples for SEM, the nanoparticles were redispersed in ethanol, and the solution of nanoparticles was drop-casted onto the surface of clean silicon wafers. After vacuum-drying for at least an hour, the samples were ready for SEM analysis.

**Figure 3:**
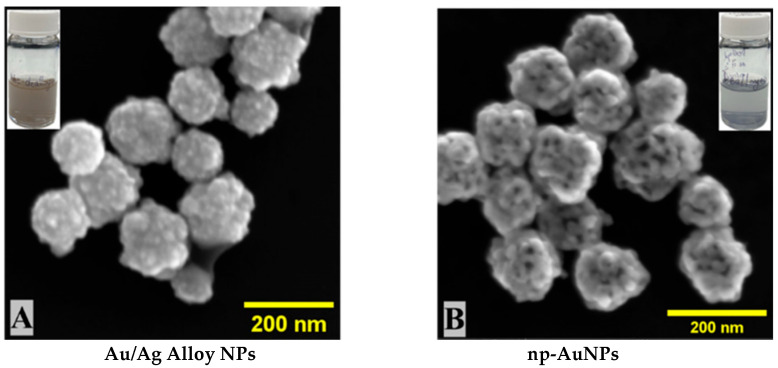
SEM images of (**A**) Au:Ag(1:3) alloy nanoparticles and (**B**) nanoporous gold nanoparticles (np-AuNPs(1:3)).

The gold (III) and silver (I) ions were co-reduced by ascorbic acid to their respective metals, from which the alloy nanoparticles were formed in the form of their interconnected ligament structure. During the dealloying process, the silver (Ag) was selectively removed from the alloy, leading to the formation of pores inside ligaments of gold (Au). Nanoporous gold nanoparticles (np-AuNPs) were successfully formed with the ligaments and pores clearly visible inside the nanoparticles through SEM imaging (Figure 3). The SEM images of alloy nanoparticles and nanoporous gold nanoparticles (np-AuNPs) are shown in Figure 3. The images in the inset are the real images of these colloidal nanoparticles in solution form. The SEM analysis further shows that these alloy and nanoporous nanoparticles possess a rough surface morphology with almost spherical shapes.

The energy-dispersive X-ray spectroscopy (EDS) was performed using ChemiSEM technology incorporated into the SEM to analyze the elemental composition of these nanoparticles.

**Figure 4:**
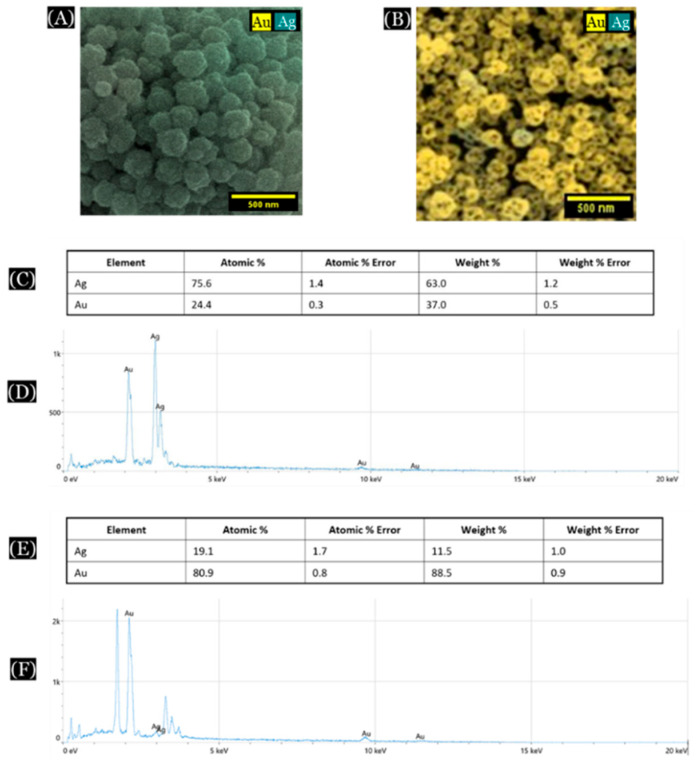
SEM/EDS analysis of Au/Ag alloy nanoparticles and nanoporous gold nanoparticles (np-AuNPs). (**A**,**B**) are color SEM images of the alloy nanoparticles and np-AuNPs, respectively; (**C**) and (**D**) show the elemental composition and EDS spectra of the alloy nanoparticles, respectively; and (**E**,**F**) show the elemental composition and EDS spectra of the np-AuNPs.

One instance of EDS analysis is discussed here (Figure 4). In this case, a 1:3 molar ratio of Au:Ag (25% of Au) was initially used to form the Au/Ag alloy nanoparticles, which was confirmed by EDS analysis of the alloy nanoparticles [Figure 4A,C,D]. After dealloying of these alloy nanoparticles, most of the silver was removed from the structure, leaving pores inside the nanoparticles leading to the formation of np-AuNPs [Figure 4B,E,F]. However, the complete removal of silver was still not observed.

### 3.3. TEM Analysis

**Figure 5:**
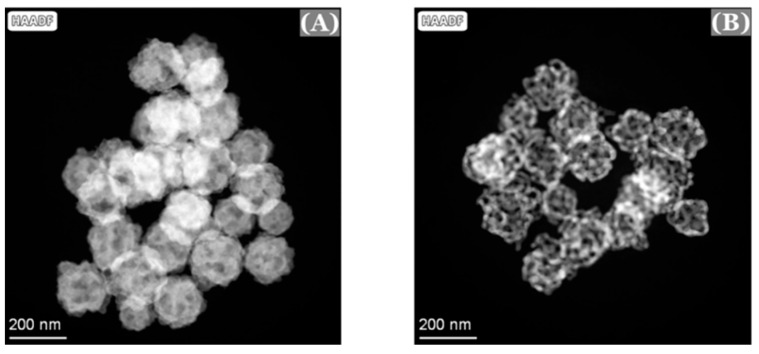
TEM images of Au/Ag alloy nanoparticles (**A**) and nanoporous gold nanoparticles (**B**).

Transmission electron microscopy (TEM) and scanning transmission electron microscopy (STEM) were also used to confirm the formation of these nanoporous gold nanoparticles (np-AuNPs). The TEM and STEM high-angle annular dark-field (HAADF) imaging were conducted on a Thermo Fisher Spectra 300 instrument operated at 300 kV. The STEM-EDS elemental mapping was acquired using the Spectra 300 with Super-X EDS technology. The Au/Ag alloy nanoparticles without pores and the interconnected ligament structures in the nanoporous gold nanoparticles (np-AuNPs) with clear pores were evident from the TEM images (Figure 5).

**Figure 6:**
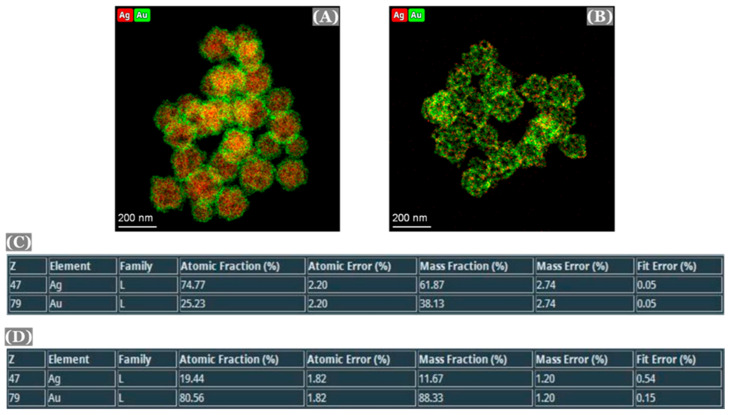
Color TEM images of Au:Ag(1:3) alloy nanoparticles (**A**), np-AuNPs (**B**), and elemental composition of the alloy nanoparticles and np-AuNPs(1:3) [(**C**,**D**), respectively].

STEM-EDS was performed to verify the elemental composition of the nanoparticles (Figure 6). The composition of the nanoparticles before and after the dealloying was observed to be in good agreement with the SEM/EDS results.

**Figure 7:**
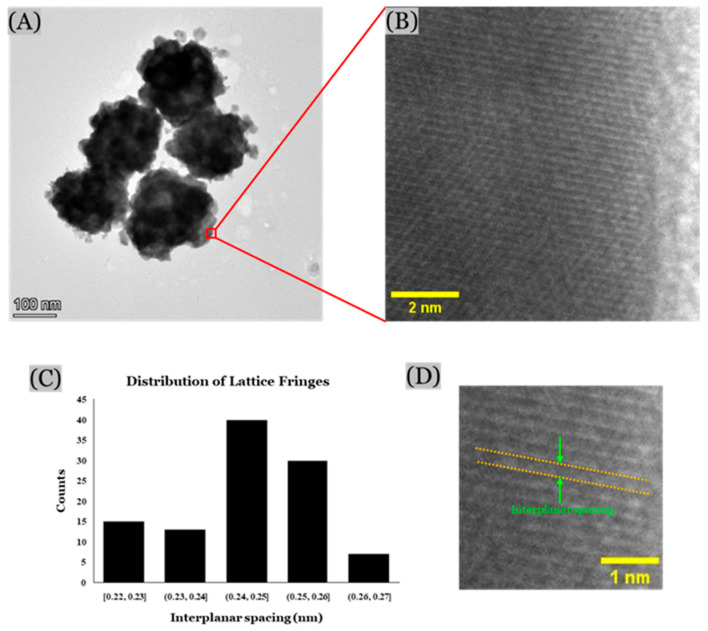
High-resolution TEM images: (**A**) TEM image of Au/Ag alloy nanoparticles, (**B**) magnified view, and (**C**) histogram showing distribution of interplanar spacing between lattice fringes (ImageJ software), and (**D**) zoomed view showing interplanar spacing.

The high-resolution TEM images of the nanoparticles were obtained using the Ceta-camera of the TEM. The size and morphology of the alloy nanoparticles analyzed with this technique were similar to those from the SEM images. However, the atomic-level resolution of this technique was able to provide more details on the lattice distribution of the nano-sized gold and silver ligaments (Figure 7). Nanocrystalline structures of gold and silver nanoparticles were observed with approximately 0.24 nm interplanar spacing between the lattice fringes, which represent their face-centered cubic (FCC) structure with (111) plane [53,54,55,56,57]. This further confirms the formation of Au/Ag alloy nanoparticles.

### 3.4. DLS Analysis

**Figure 8:**
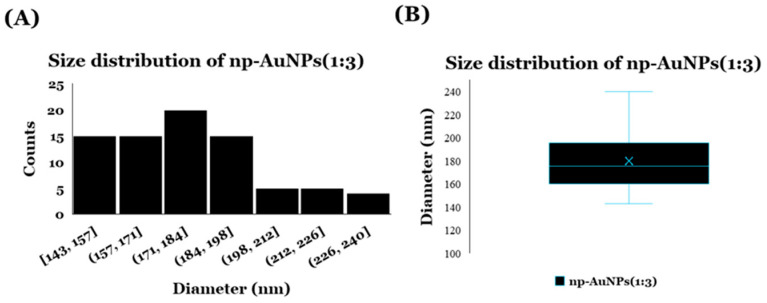
DLS data showing size distribution of np-AuNPs(1:3): (**A**) histogram and (**B**) box plot.

The dynamic light scattering (DLS) analysis was performed with a DynaPro Titan Instrument (Wyatt Technology, Santa Barbara, CA, USA) to observe the size distribution of these np-AuNPs prepared using an Au-to-Ag molar ratio of 1:3 Figure 8. A total of 35 μL of sample was placed in a quartz cuvette, and the light scattering intensity was collected at a 90° angle using an acquisition time of 5 s. The mean diameter was calculated from mean hydrodynamic radius of the particles, which was obtained using Dynamics software (version 6.12.03) [58]. The average diameter of the np-AuNPs was observed to be 186 ± 31.69 nm. The size distribution is shown in Figure 8.

### 3.5. Sizes of np-AuNPs

**Figure 9:**
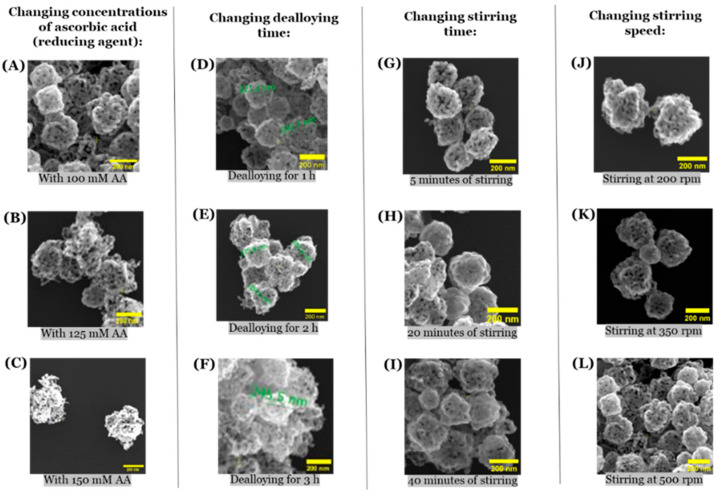
SEM images of np-AuNPs showing the effects of different parameters on the size of the np-AuNPs. (**A**–**C**) are the SEM images of np-AuNPs obtained using 100 mM, 125 mM, and 150 mM of reducing agent (ascorbic acid), respectively; (**D**–**F**) are the SEM images of np-AuNPs formed during 1h, 2h, and 3h of dealloying time, respectively; (**G**–**I**) are the SEM images of np-AuNPs obtained after stirring for 5, 20, and 40 min, respectively; and (**J**–**L**) are the SEM images of np-AuNPs formed with stirring speeds of 200, 350, and 500 rpm (revolutions per minute).

Considering that the size-specific advantages of np-AuNPs depend on the field of application, the next important effort was to tune their sizes. For this purpose, we tried different approaches, such as changing the concentration of reducing agent (ascorbic acid), changing the dealloying time with nitric acid, changing the stirring time, and changing the stirring speed (Figure 9). We did not notice any significant change in the sizes of these np-AuNPs during these experiments. For these experiments, we used a 1:3 Au:Ag ratio for reference, and the size of the np-AuNPs remained similar, around a value of approximately 200 nm.

Afterwards, when we changed the gold-to-silver (Au:Ag) ratio before the alloy nanoparticle formation, we finally observed a significant change in the sizes of np-AuNPs.

**Figure 10:**
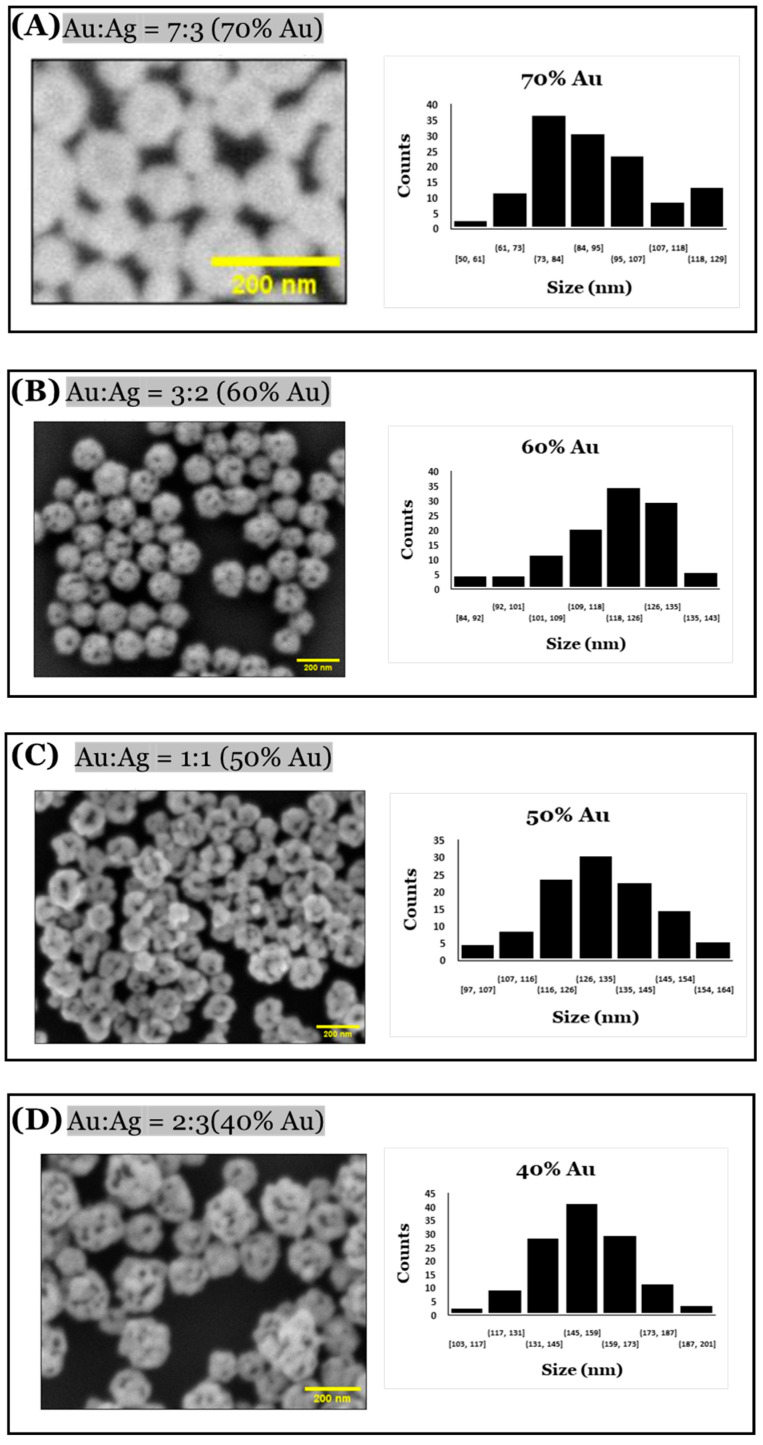

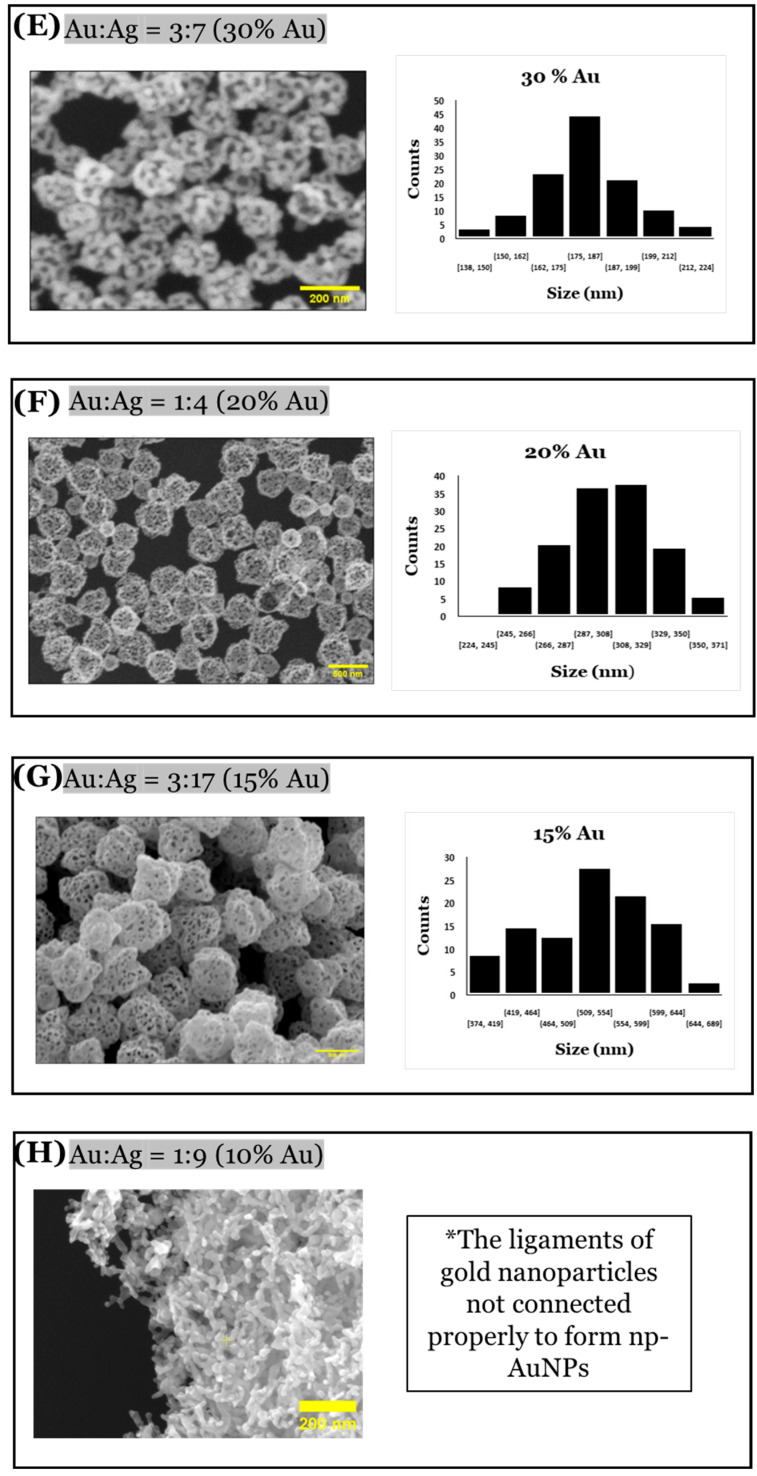
SEM images of np-AuNPs formed using different Au:Ag ratios and histograms showing their respective size distributions. The numbers under each bar in the bar charts represent ranges of dimensions in nm, and counts is the number of observed pores in that range.

**Figure 11:**
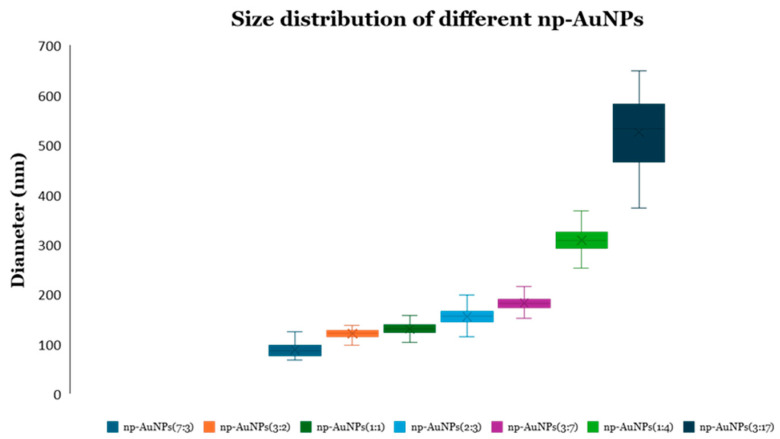
Box plot showing the size distributions of various np-AuNPs. The ratios in parentheses specify the Au:Ag ratio from which the alloy nanoparticles were prepared.

A significant change in sizes and pores of the np-AuNPs was observed with a change in the molar ratio between the gold and silver (Figure 10). With an Au:Ag ratio of 7:3, i.e., using 70% gold in the alloy nanoparticles, the size of the particles was observed to be smaller, having an average diameter around 90 nm; however, the pores were not visible clearly inside the nanoparticles. Using an Au:Ag ratio of 3:2, i.e., with 60% gold composition, np-AuNPs with an average size of around 120 nm were observed, with clear pores inside the nanoparticles. A gradual increase in the size of the np-AuNPs and their pores was observed with a decrease in the gold percentage, and the silver percentage was increased until there was a 15% gold composition. With 15% gold in the alloy nanoparticles, bigger np-AuNPs with an average diameter of around 530 nm were observed, with clearer and wide-open pore structures. The gold ligaments were more clearly visible in these np-AuNPs. But, when we used a Au:Ag ratio of 1:9, i.e., with 10% gold, the gold ligaments were not connected to each other to form the np-AuNPs structure. The trend in the change in the sizes of these np-AuNPs can be clearly observed in the box plot shown in Figure 11. This illustrates the importance of gold and silver composition in the preparation of np-AuNPs. The observed results are summarized in Table 1.

Numerous theories have been proposed to explain the nucleation and growth of nanoparticles [59]. One widely accepted model, the LaMer mechanism [60], outlines three key stages: First, there is a rapid rise in the concentration of free monomers. This is followed by the nucleation of these monomers, and finally, a diffusion-controlled growth of the nuclei, ultimately leading to nanoparticle formation. The Ostwald ripening [61] theory explains nanoparticle growth based on the solubility and surface energy of the smaller particles, particularly the monomers. According to this theory, smaller, more soluble particles with a higher surface energy tend to dissolve back into the solution, enabling the growth of larger particles. Another model, the Finke–Watzky two-step mechanism [62], suggests that nucleation and growth occur simultaneously. Additionally, coalescence and intraparticle ripening are two other commonly accepted mechanisms that describe nanoparticle growth [63,64,65]. Despite the variations among these models, they all emphasize two fundamental steps in nanoparticle formation: (i) nucleation and (ii) growth.

Many studies concur that there are similar mechanisms for the synthesis of gold (Au) and silver (Ag) nanoparticles, which generally follow two primary stages: (i) reduction and nucleation, and (ii) growth through coalescence of the nuclei [66]. Although both metals follow similar formation principles, the synthesis of silver nanoparticles is influenced by additional factors, leading to differences in their growth pathways. Gold nanoparticles tend to form through a faster, single-step coalescence process. In contrast, the growth of silver nanoparticles is slower, due to factors such as changes in surface chemistry [66], the back-oxidation of Ag^0^ to Ag^+^ through hydrolysis, the lower reduction potential of Ag^+^ compared to [AuCl_4_]^−^ [67], and the low solubility of AgCl [68]. These variables are particularly important in the formation of gold/silver (Au/Ag) alloy nanoparticles and, eventually, nanoporous gold nanoparticles (np-AuNPs).

In a solution containing both Au^3^^+^ and Ag^+^ precursor ions, the slower reduction rate of Ag^+^ leads to an initial surplus of Au atoms [67,69]. During nucleation, this excess results in the formation of Au-rich nuclei. As the reaction proceeds, more Ag atoms are incorporated into the growing nanoparticles, primarily contributing to their enlargement. This process can be expected to lead to the formation of larger Au/Ag alloy nanoparticles, with a higher composition of Ag in the alloy nanoparticles. A core–shell structure with a Au-rich core and Ag-rich shell can be expected from this mechanism. Over time, atomic rearrangement leads to the formation of a homogeneous Au/Ag alloy.

The final stage in forming nanoporous gold nanoparticles (np-AuNPs) via the dealloying method involves two main processes: (i) dissolution and (ii) diffusion [70]. First, the less noble Ag atoms are selectively dissolved, leaving behind vacancies in the crystal lattice. Subsequently, the Au atoms reorganize into clusters. These developments are followed by a diffusive redistribution of both the Au atoms and the generated vacancies across adjacent lattice sites, ultimately creating a three-dimensional porous structure.

### 3.6. Effects of Dealloying Time

We also studied the effects of dealloying time on the formation of np-AuNPs. No significant changes in the size of the np-AuNPs were observed by changing the time of dealloying. However, the EDS analysis of elemental composition showed that less silver was present inside the np-AuNPs with an increase in the dealloying time. As one example, in the np-AuNPs prepared by using a molar ratio of Au:Ag of 1:3, the 19.1% (i.e., 11.5% *w*/*w*) silver content present in the sample after 4 h of dealloying was reduced to 7.6% (i.e., 4.3% *w*/*w*) after 10 h. The results for the EDS data at different dealloying times are shown in Table 2.

Residual silver may influence the biocompatibility, electrochemical performance, stability, and selectivity of a biosensor. While silver nanoparticles have been extensively researched for their biomedical applications, numerous studies have reported their potential cytotoxic effects, depending on factors such as their particle size, concentration, and surface functionalization [71]. Moreover, the stability of a biosensor over time could be compromised by the presence of higher silver content, as it is less stable than gold. To address these issues, we aimed to reduce the silver content in the np-AuNPs by increasing the dealloying time to overnight. Furthermore, the use of analyte-specific antibody and a blocking agent was expected to enhance the selectivity and sensitivity by minimizing the risk of potential false positives.

### 3.7. UV-Vis Spectroscopic Analysis and Surface Plasmon Resonance

UV-Vis spectroscopy can provide valuable insights into the properties of nanoparticles, including their size and shape. It can also detect the localized surface plasmon resonance (LSPR) spectra of nanoparticles, which gives the characteristic absorption peaks corresponding to the size and shape of the nanoparticles. In this work, we used UV-Vis spectroscopy for the characterization of Au/Ag alloy nanoparticles and np-AuNPs that we synthesized.

**Figure 12:**
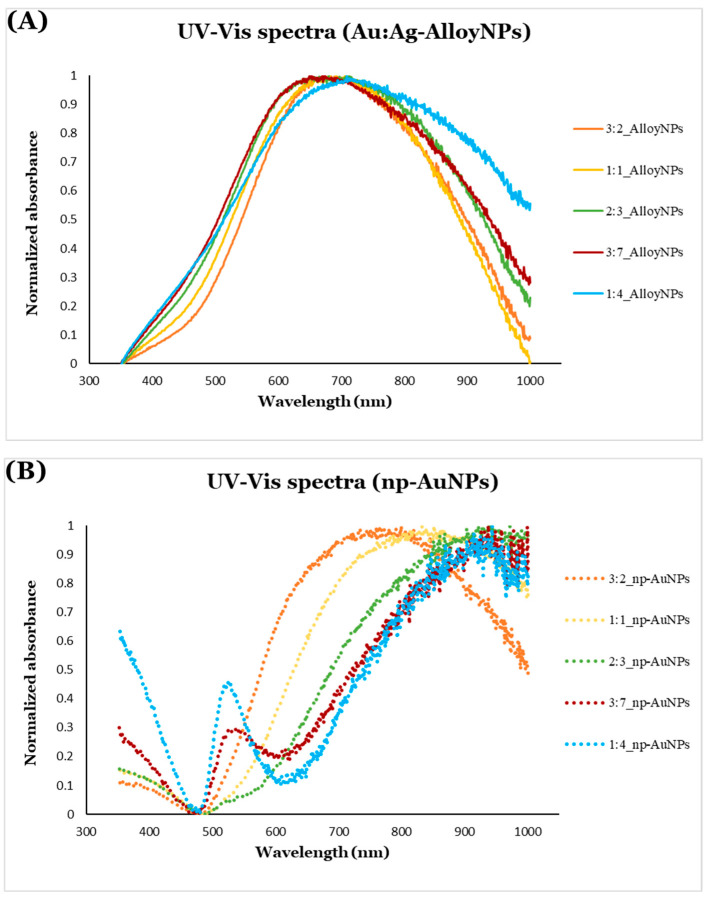
UV-Vis extinction spectra of (**A**) Au/Ag alloy nanoparticles (solid lines) and (**B**) np-AuNPs (dotted lines) of different sizes.

The UV-Vis extinction spectra of Au/Ag alloy nanoparticles (solid lines in Figure 12A) reveal peaks due to the combined effects of absorption and scattering within the visible range (approximately 600 nm to 650 nm). Minimal changes are observed in the absorption spectra as the alloy nanoparticle size varies; however, as the alloy nanoparticle size increases, a slight red shift in the absorption peaks occurs. For particles of this size, scattering would be the dominant contribution to the recorded spectra. Separating the effects of absorbance and scattering would require use of an integrating sphere [72]. The features of the spectra shown in Figure 12 are similar to those reported experimentally by Wrigglesworth [73] and from calculations by Ru and coworkers [74,75].

In contrast, a more pronounced red shift is seen for the np-AuNPs (dotted lines in Figure 12B) compared to the alloy nanoparticles. The extinction peaks of the np-AuNPs are in the near-infrared (NIR) region (around 750 nm to 1000 nm). A significant, gradual red shift is observed for the np-AuNPs as their size increases, which can be attributed to the broader open pore structures in the np-AuNPs compared to the solid packed arrangement in the alloy nanoparticles. Interestingly, larger np-AuNPs also exhibit additional absorption peaks near 525 nm, a feature almost absent in smaller np-AuNPs. It is possible that these peaks represent localized surface plasmon absorbance due to the internal structure of gold ligaments that varies with the particle size. Smaller np-AuNPs have smaller pores and a more tightly packed ligament structure, while larger np-AuNPs feature larger pores and a more open ligament structure, which may account for the absorption peak around 525 nm that is typical of Au nanoparticles or the transverse mode of Au nanorods. This effect is particularly noticeable in the larger particles, such as those with Au:Ag molar ratios of 3:7 and 1:4, likely due to their open ligament structures. This indicates that the larger np-AuNPs exhibit surface plasmon resonance in both the visible and NIR regions, offering an additional advantage due to their open pore structures and larger surface area.

Given the very small ligament size in the np-AuNPs, it is likely that Rayleigh light scattering also occurs. This effect may contribute to the increased spectral intensity observed in the np-AuNPs, particularly in the larger particles with more open ligament structures, toward the blue end of the spectrum. Similarly, Mie scattering is likely responsible for the features near the red end of the spectrum, as light interacts with the larger-sized np-AuNPs, influencing their apparent absorption spectra. These scattering phenomena are more prominent in larger np-AuNPs, such as np-AuNPs(1:4) and np-AuNPs(3:7), due to their greater size and more widely opened ligament networks [76,77]. The absorbance, scattering, and plasmonic behavior of these particles awaits further investigation by experiment and calculation.

### 3.8. Application of np-AuNPs in LPS Biosensing

Electrodes modified with nanoparticles show significant promise for electrochemical sensing, thanks to their increased surface area, superior electrocatalytic activity, and enhanced electron transfer capabilities that contribute to greater sensitivity, selectivity, and stability compared to unmodified electrodes. These benefits are further amplified in nanoporous gold nanoparticles (np-AuNPs), which combine the advantages of nanoscale dimensions with porous architecture. The larger surface area of np-AuNPs offers more active sites for analyte adsorption, leading to stronger electrochemical responses. Their open porous structure, presence of low-coordinated atoms, and enhanced surface area also promote faster and more efficient electron transfer, resulting in a lower detection limit. Additionally, np-AuNPs can be easily functionalized with specific groups to target the desired analytes, further improving their selectivity. Their structure also provides a stable matrix for attaching biomolecules, enhancing the overall stability of a biosensor. Taking advantage of these exceptional features, we propose using an np-AuNPs-modified ITO-coated glass electrode for the sensitive detection of lipopolysaccharide (LPS).

#### 3.8.1. Proposed Biosensing Platform for LPS Detection

**Figure 13:**
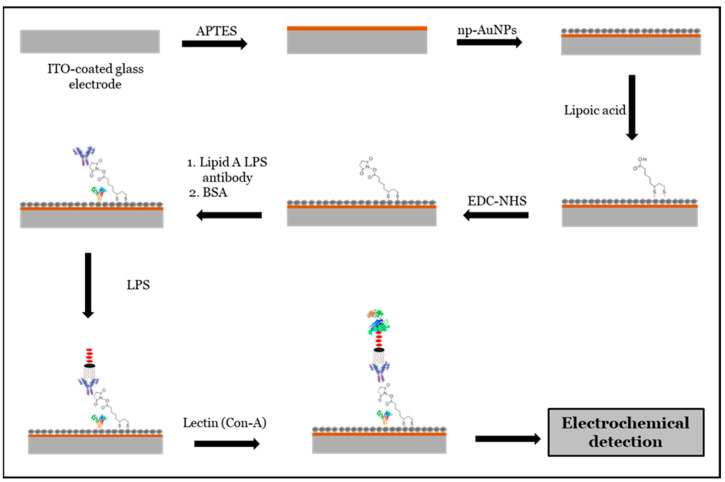
Schematic representation of proposed mechanism for LPS biosensing.

In this approach, a clean ITO-coated glass electrode surface was initially treated with (3-aminopropyl)triethoxysilane (APTES) to enable the attachment of nanoporous gold nanoparticles (np-AuNPs). After modifying the electrode with np-AuNPs, it was further functionalized with lipoic acid, followed by EDC-NHS chemistry to immobilize a layer of lipid A LPS polyclonal antibodies, which specifically recognize the lipid A region of LPS. Bovine serum albumin (BSA) was used to prevent the non-specific binding of the antibodies. To enhance the electrochemical signal, concanavalin A (Con A), a lectin with a high affinity for carbohydrate structures, was introduced to bind to the carbohydrate components of LPS, forming a sandwich-type assay. Detection of LPS was then carried out using electrochemical impedance spectroscopy (EIS). A schematic illustration of this proposed LPS biosensing route is shown in Figure 13.

#### 3.8.2. SEM Characterization of Modified Electrode

**Figure 14:**
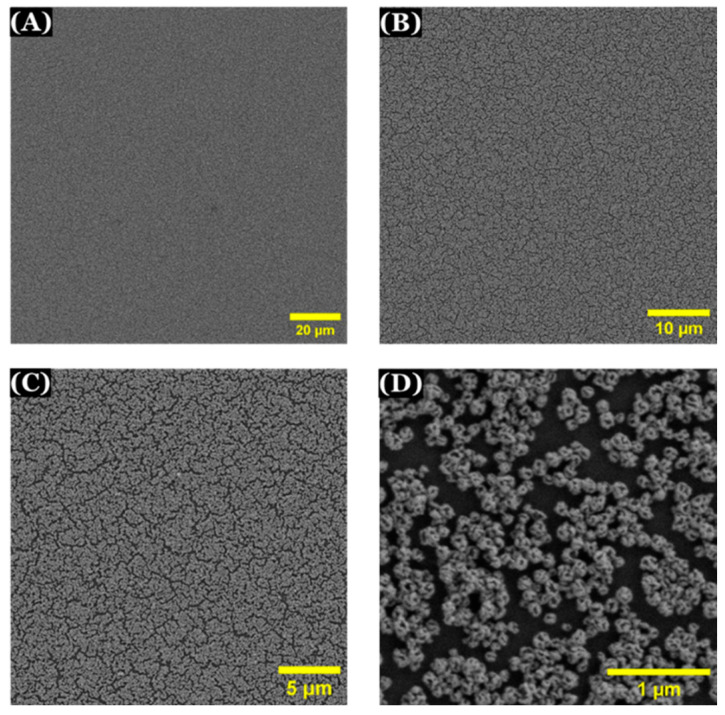
SEM images of np-AuNPs-modified electrode with different magnifications (**A**) 2000×; (**B**) 5000×; (**C**) 10,000× (**D**) 65,000×.

Scanning electron microscopy (SEM) was used to observe the surface of np-AuNPs-modified electrode. A well-distributed layer of np-AuNPs was observed to be immobilized on the surface of APTES-functionalized ITO-coated glass electrode (Figure 14).

#### 3.8.3. Electrochemical Characterization of Modified Electrode

Various electrochemical techniques are available to investigate the surface characteristics of modified electrodes, offering valuable information about their behavior and properties. Cyclic voltammetry (CV) and electrochemical impedance spectroscopy (EIS) are among the most widely used techniques. These techniques provide insights into redox reactions, charge/electron transfer mechanisms, diffusion processes, and other electrochemical phenomena at the electrode/electrolyte interface. In this study, CV and EIS were employed to examine the redox behavior and charge transfer processes on ITO-coated glass electrodes modified with nanoporous gold nanoparticles (np-AuNPs).

##### Cyclic Voltammetry (CV) of the Modified Electrode

Cyclic voltammetry (CV) is a powerful electrochemical technique in which the electrode potential is swept while the resulting current is recorded [78]. This method yields valuable insights into redox reactions, electron transfer dynamics, and adsorption/desorption behaviors. By examining the shape and position of the peaks on the CV curve, one can assess the reaction reversibility, detect adsorbed species, and estimate the electroactive surface area [78]. In this study, CV was carried out using a three-electrode system composed of a modified electrode as the working electrode, an Ag/AgCl electrode as the reference electrode, and a platinum wire as the counter electrode. The redox probe Fe(CN)_6_^4−/3−^ was used, with a scan rate of 0.1 V/s and an amplitude of 5 mV, over a potential window ranging from −0.2 to +0.6 V.

**Figure 15:**
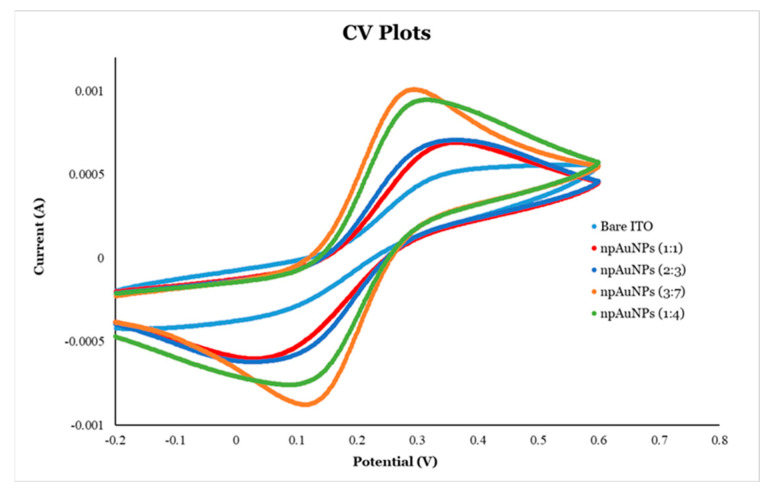
Cyclic voltametric (CV) analysis of ITO-coated glass electrodes modified with different sizes of np-AuNPs.

Cyclic voltammetry (CV) was employed to investigate the electrochemical behavior of electrodes modified with nanoporous gold nanoparticles (np-AuNPs) of varying sizes. Compared to bare ITO-coated glass electrodes, all the modified electrodes exhibited sharper and more symmetrical redox peaks with an increased current, indicating enhanced electron transfer capabilities. Among these, the electrodes modified with larger np-AuNPs demonstrated superior electrochemical performance (Figure 15 and Table 3). Specifically, those prepared with np-AuNPs synthesized using Au:Ag ratios of 1:4 [np-AuNPs(1:4)] and 3:7 [np-AuNPs(3:7)] showed more pronounced peaks and higher peak currents than those modified with smaller np-AuNPs, such as np-AuNPs(2:3) and np-AuNPs(1:1). Additionally, a reduction in the peak separation (∆E), the potential difference between anodic and cathodic peaks, was observed for the electrodes with larger np-AuNPs. The anodic-to-cathodic peak current ratios (|I_p,a_/I_p,c_|) were also closer to unity for these electrodes, indicating faster electron transfer kinetics and more reversible redox reactions compared to those modified with smaller np-AuNPs.

##### Electrochemical Impedance Spectroscopy (EIS) of the Modified Electrode

Electrochemical impedance spectroscopy (EIS) is a highly effective technique for measuring a circuit’s impedance, its resistance to alternating current (AC), across a range of frequencies. In biosensing, EIS is extensively used for its exceptional sensitivity and precision in detecting interfacial changes at electrode surfaces during bio-recognition events. Impedance accounts for both the amplitude and phase shift between voltage and current, and is expressed as a complex number with real (Z_re_) and imaginary (Z_im_) components. The behavior of impedance is commonly analyzed using Nyquist and Bode plots. In Nyquist plots, −Z_im_ is plotted against Z_re_, with the semicircle’s diameter in the plot corresponding to the charge-transfer resistance (R_ct_), and the linear portion indicating diffusion-limited processes. Bode plots, on the other hand, depict the impedance magnitude and phase angle as functions of frequency. The integration of nanomaterials, such as nanoparticles, nanotubes, nanowires, and nanocomposites, has been shown to enhance the performance of EIS-based biosensors by accelerating electron transfer, boosting catalytic activity, and enabling more effective biomolecule immobilization, ultimately improving sensitivity, accuracy, and reliability [79,80,81].

In this study, the electrochemical properties of np-AuNPs-modified electrodes were investigated using electrochemical impedance spectroscopy (EIS). A three-electrode system consisting of a working electrode (modified electrodes), reference electrode (Ag/AgCl electrode), and a counter electrode (platinum wire) were used in the electrochemical cell. Fe(CN)_6_^4−/3−^ served as the redox probe. A sinusoidal potential of 0.2 V (vs. reference electrode) with an amplitude of 10 mV was applied to the electrochemical cell across a frequency range of 100 kHz to 0.1 Hz. The data were collected at a rate of 12 points per frequency-decade.

**Figure 16:**
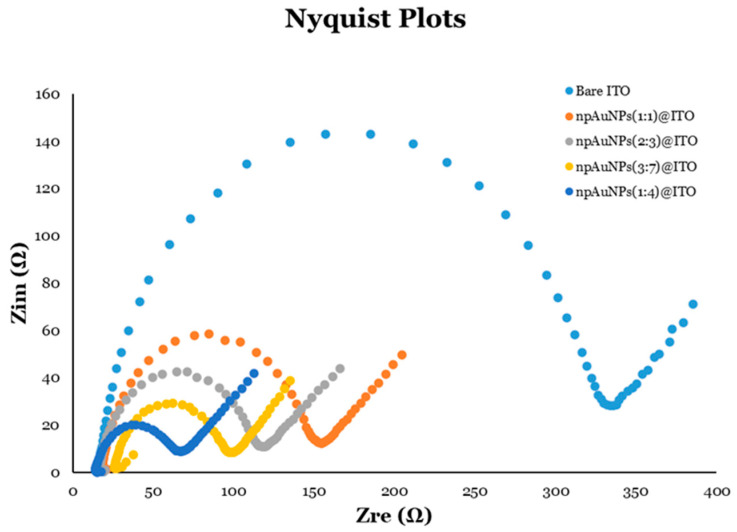
Nyquist plots for different electrodes with or without modification using different sizes of np-AuNPs.

Nyquist plots were generated and studied for bare ITO-coated glass electrodes and their modifications with different sizes of np-AuNPs (Figure 16). The semicircle portion of the Nyquist plot is easily observed, and provides valuable information regarding charge-transfer resistance (R_ct_) of the electrodes. A larger semicircle corresponds to a higher R_ct_, indicating slower electron transfer, while a smaller semicircle signifies a lower R_ct_ and thus more efficient electron transfer. From the Nyquist plot in Figure 16, the electrode modified with np-AuNPs(1:4) showed the smallest semicircle (dark blue), indicating the lowest R_ct_ and, consequently, the most efficient electron transfer and best electrode performance among the samples tested.

Electron transfer kinetics are often linked to the surface area and accessibility of the active sites, both of which are influenced by the ligament connectivity and the size of the pores [12,13,82,83,84,85]. The modified electrodes containing np-AuNPs with a more interconnected, open ligament structure and larger pores demonstrated faster electron transfer kinetics, likely due to their increased surface area and more efficient conductive pathways. In contrast, the np-AuNPs with denser ligament structures, resulting in smaller pores, had a reduced accessible surface area and fewer active sites, which could have hindered the electron transfer processes. This was supported by the electrochemical data, including the cyclic voltammetry (CV) and electrochemical impedance spectroscopy (EIS) experiments. Higher peak currents and lower impedances were observed for the np-AuNPs with larger pores and widely interconnected ligaments (e.g., modified electrodes with np-AuNPs(1:4) and np-AuNPs(3:7)), while the np-AuNPs with smaller pores and more densely connected ligaments (e.g., modified electrodes with np-AuNPs(1:1) and np-AuNPs(2:3)) exhibited lower peak currents and higher impedances.

An SEM analysis was performed to examine the shape, size, and porosity of the np-AuNPs, ensuring consistency and reproducibility during the scaling process. Similar SEM results were observed for the np-AuNPs synthesized at both small and large scales. For the electrochemical testing, initial cyclic voltammetry (CV) and electrochemical impedance spectroscopy (EIS) measurements were carried out using smaller batches of np-AuNPs. Subsequently, the electrochemical experiments were repeated with np-AuNPs synthesized in larger batches, yielding comparable electrochemical responses in both cases.

#### 3.8.4. Electrochemical Impedance Spectroscopy (EIS) for LPS Detection

Following the microscopic, spectroscopic, and electrochemical characterization of the different np-AuNPs-modified electrodes, the np-AuNPs(1:4)-modified ITO-coated glass electrode was finally selected for use in LPS biosensing based on its highly porous structure, accessible interconnected ligaments, and better electrochemical performance. The electrochemical impedance spectroscopy (EIS) was used for the detection of a smooth (wild-type) LPS from *Escherichia coli* O55:B5 (Millipore Sigma), purified by phenol extraction.

##### Procedure for Electrode Modification and LPS Assay

As mentioned in the previous section, the surface of clean ITO-coated glass electrodes was modified with np-AuNPs(1:4) using an O_2_-plasma treatment followed by APTES functionalization. The O_2_-plasma treatment helped remove organic contaminants from the electrode surface, making the hydroxide bonds more available for APTES [40,41]. APTES is a well-known linker used to immobilize gold nanoparticles through the electrostatic interactions between its amine groups and gold nanoparticles [42,43,44]. The np-AuNPs(1:4)-modified electrodes were then treated with 10 mM lipoic acid (in ethanol) overnight and rinsed with ethanol and acetonitrile at least three times each. This was followed by EDC-NHS chemistry to activate carboxylic groups of lipoic acid to attach a lipid A of LPS-specific antibody. Lipoic acid is widely used in various applications, including biosensing, due to its ability to interact with various biological molecules [45,46]. The lipoic acid-treated electrodes were then immersed in a solution containing a mixture of 5 mM EDC and 5 mM NHS for at least 5 h. Then, the electrodes were rinsed with acetonitrile at least three times, dried using N_2_ gas, and again rinsed with 10 mM PBS buffer (pH 7.2). After this step, the modified electrodes were left immersed overnight in a 10 mL solution containing 80–100 μg of lipid A LPS polyclonal antibody. The lipid A LPS polyclonal antibody was used for binding lipid A segment of LPS molecules with specificity and accuracy. The antibody-functionalized electrodes were then treated with a blocking buffer, 1% BSA in 10 mM PBS buffer (pH 7.2) (i.e., 1 g BSA in 100 mL of buffer), for at least 30 min to prevent non-specific binding of the antibody by competing for the binding sites, thus reducing background noises and improving the signal-to-noise ratios [47,48]. The modified electrodes were then rinsed with 10 mM PBS (pH 7.2) and stored at 4–8 °C in 10 mM PBS buffer (pH 7.2) for further use as the biosensing platforms.

For LPS biosensing, different solutions with different concentrations of LPS were prepared by dissolving a wild-type LPS from *Escherichia coli* O55:B5 in 10 mM PBS buffer (pH 7.2), ranging from 1 ng/L to 1000 ng/L. A 1g/L concanavalin A (con A) solution was prepared in a binding buffer (10 mM Tris-buffer containing 100 mM NaCl, 1 mM CaCl_2_, and 1 mM MnCl_2_) for making a sandwich assay by binding with the carbohydrate (O-antigen) portion of LPS. For sensing purposes, the modified electrodes were immersed in the solution with different concentrations of LPS for 1 h, rinsed with 10 mM PBS buffer, and again treated with 1 g/L con A solution for another 1 h. The electrodes were ready for electrochemical measurements after rinsing once again with buffer solution followed by Milli-Q water and drying under N_2_ gas.

**Figure 17:**
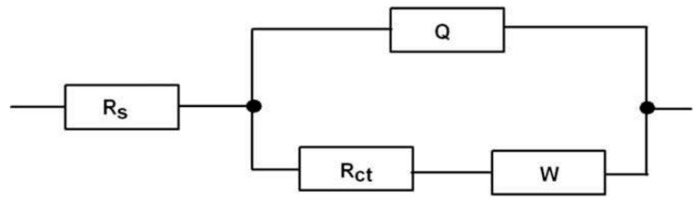
The Randles equivalent circuit model, R(Q(RW)), used in this study [86].

**Figure 18:**
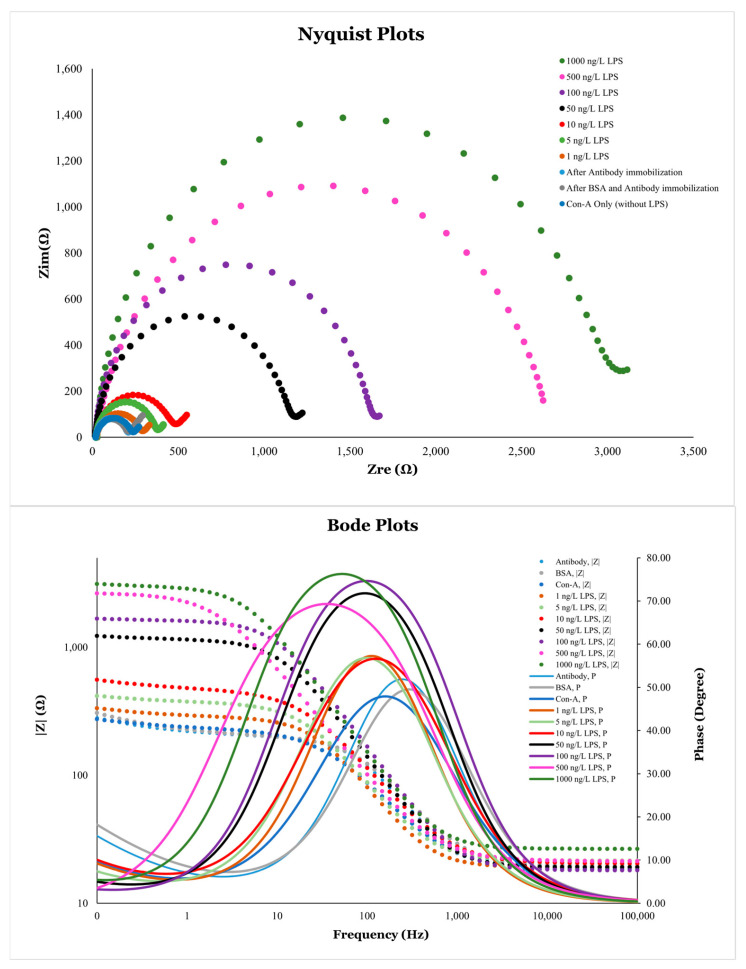
Nyquist plots (**A**) and Bode plots (**B**) for different analytes immobilized on the surface of np-AuNPs(1:4)-modified electrode.

Electrochemical impedance spectroscopy (EIS) was performed using a similar three-electrode system and redox probe as mentioned earlier. A simplified Randles equivalent circuit model, R(Q(RW)) (Figure 17) [86], was used for the analysis of data using ZSimpWin software. Different Nyquist plots were obtained for the different analytes immobilized on the electrode surface (Figure 18). In this model of Randles equivalent circuit, R_s_ represents the solution resistance; Q is a constant phase element (CPE), which represents the double-layer capacitance and its non-ideal behavior; R_ct_ represents the charge-transfer resistance (occurring at the electrode surface); and W, known as the Warburg impedance, is related to mass transport of species at the electrode surface. In this type of circuit, the impedance can be expressed as [79]

(1) $$Z\left(\omega \right)=R_{s}+\frac{R_{ct}}{1+{\left(\omega R_{ct}Q\right)}^{2}}-j\frac{\omega R_{ct}^{2}Q}{1+{\left(\omega R_{ct}Q\right)}^{2}}$$

where ‘j’ is an imaginary number (j = $\sqrt{\left(-1\right)}$).

Another important equation related to this circuit, showing the relationship between the real and imaginary parts of the impedance, is given by [79]

(2) $$(Z_{re}\;-\;R_{s}\;-\;\frac{R_{ct}}{2}{)}^{2}\;+\;{\left(Z_{\mathrm{im}}\right)}^{2}\;=\;{\left(\frac{R_{ct}}{2}\right)}^{2}$$

As a result, in the Nyquist plot, the variation in Z_re_ relative to Z_im_ produces a circular plot with a radius of R_ct_/2, with its center at Z_re_ = R_s_ + R_ct_/2 and −Z_im_ = 0.

Electrochemical impedance spectroscopy (EIS) is a very sensitive technique for detecting the change in the charge-transfer resistance (*R_ct_*) on the surface of an electrode, which was clearly observed in this study. Nanoscale changes in the analyte concentration on the surface of modified electrodes had very significant effects on its *R_ct_* values. Changing the LPS concentration from 1 ng/L to 1000 ng/L showed very significant changes in the EIS response, which can be observed from the Nyquist plots with the naked eye by analyzing its semicircle portion (Figure 18A). Using Equation (4), at very high frequencies, ω → ∞ and *Z = R_s_*, while at very low frequencies, ω → 0 and *Z = R_s_ + R_ct_*. These boundaries conditions can be observed on the Nyquist plots as the first (starting) and second (ending) points of the semicircle on the *x*-axis having real impedance. The change in the R_ct_ values is attributed to the resistance caused by the analytes on the electrode surface. The higher R_ct_ values correspond to the greater resistance produced by the analytes inhibiting the electron transfer process and vice versa.

The Bode plots were also analyzed for these analytes immobilized on the surface of modified electrodes. In Bode plots, the direct variations in the amplitude of impedance (|Z|) and phase shift (φ) as a function of frequency can be observed. The frequency-dependent data is not directly visible in Nyquist plots. The change in the impedance magnitude (|Z|) with frequency (ω) can be observed as an S-shaped curve in Figure 18B. The impedance at high frequencies corresponds to solution resistance, while that near low frequencies provides information about the total resistance of the electrochemical cell. The bell-shaped curves in the Bode plot represent the variations in phase angle (φ) with frequency. The peaks of the curves indicate the presence of a double-layer capacitor in parallel to a resister in the circuit. A greater EIS response can be observed in the peak frequency range, from approximately 10 Hz to 800 Hz. The effect of different analytes on the R_ct_ can also be analyzed by observing the peak magnitudes of these bell-shaped curves.

**Figure 19:**
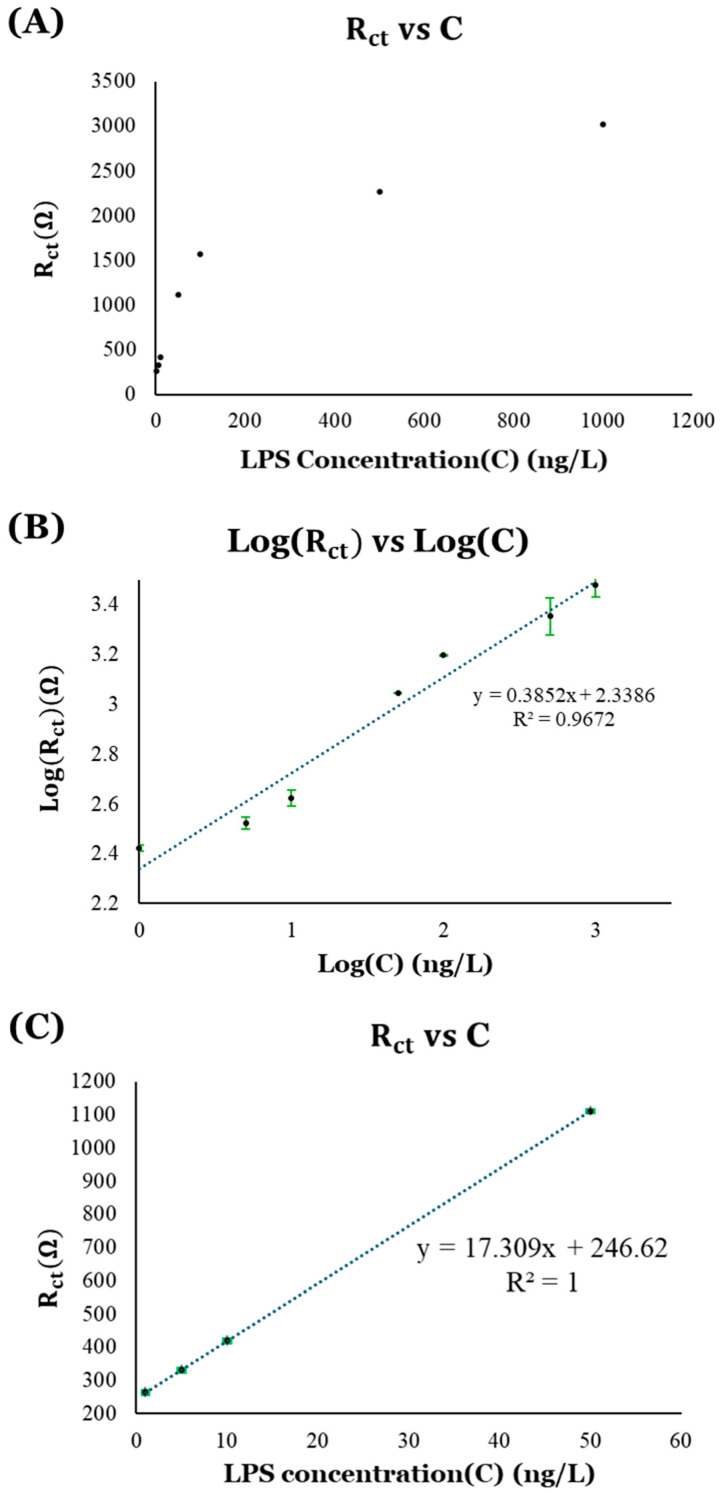
Calibration curves for LPS detection: (**A**) plot of *R_ct_* vs. LPS concentration (**C**); (**B**) logarithmic calibration curve showing Log(*R_ct_*) vs. Log(C); and (**C**) linear calibration curve using values of *R_ct_, represented by the black circles with green error bars, * near region of lower limit of detection.

The R_ct_ values from the EIS plots were obtained using ZSimpWin software (Table 4). A significant change in the R_ct_ value was observed for different analytes as well as for varying concentrations of LPS. This data was used to plot the calibration curves (Figure 19) for calculating limit of detection (LOD) and limit of quantification (LOQ) for LPS biosensing using the np-AuNPs-modified biosensing platform. As per the International Council for Harmonisation of Technical Requirements for Pharmaceuticals for Human Use (ICH) guidelines (Q2(R2), 2024), the LOD and LOQ can be expressed as follows:

(3) $$\mathrm{LOD}\;=\;\frac{3.3\sigma }{S}$$

(4) $$\mathrm{LOQ}=\frac{10\sigma }{S}$$

where σ is the standard deviation of the response and S is the slope of the calibration curve.

##### Calibration Curves for LPS Detection

First, the values of charge-transfer resistance (*R_ct_*) obtained from the EIS data were plotted against the different concentrations of LPS (C). This resulted in a logarithmic curve showing the logarithmic relation between the charge-transfer resistance (*R_ct_*) and LPS concentration (C), as depicted in Figure 19A. Considering this logarithmic relationship between the *R_ct_* and C, a calibration curve was plotted using logarithmic value of *R_ct_* (i.e., Log(*R_ct_*)) versus logarithmic value of C (i.e., Log(C)), shown in Figure 19B. The linear trendline on the Log(*R_ct_*) vs. Log(C) plot shows an R-squared (R^2^) value closer to 1 (R^2^ = 0.9672), indicating the good fit of the data. A limit of detection (LOD) of 0.7226 ng/L and a limit of quantification (LOQ) of 2.1896 ng/L were obtained using this calibration curve.

The logarithmic relationship between the values of *R_ct_* in a wide range of LPS concentrations (C), shown in Figure 19A, suggested that the *R_ct_* response is significant in the lower LPS concentration regions, where there is a linear increase in the *R_ct_* values with the LPS concentrations (C), while it appears to form a plateau near the higher LPS concentration regions, indicating saturation in the response. As a result, another calibration curve (Figure 19C) was plotted using the data near the lower range of LPS concentrations for more accuracy. In this plot of *R_ct_* vs. LPS concentrations, the linear fit of the data is better (R^2^ = 1), and produces a lower LOD (0.1244 ng/L) and LOQ (0.3768 ng/L). The values of LOD and LOQ for these sensing platforms are significantly below the LPS concentration that can be detected by our immune system.

Both the np-AuNPs and np-AuNPs-modified electrodes were stored at 2–8 °C and used for nearly two months until all the electrochemical experiments were completed. While we did not monitor their stability over specific time intervals, they performed reliably throughout the entire experimental period.

Considering the use of specific antibody for ensuring the selective and specific binding of LPS, and the use of blocking agent to prevent possible non-specific interactions on the biosensing platform, we did not conduct experiments with real samples and other possible interfering biomolecules.

## 4. Conclusions

This work resulted in a simple and efficient solution-phase synthesis of size-tunable nanoporous gold nanoparticles (np-AuNPs) with enhanced surface structures, better spectroscopic properties, and amazing electrochemical responses. This method provides an easier and straightforward way of synthesizing nanoporous gold nanoparticles in comparison to the more complex procedures that have been used in the past. These np-AuNPs were then used to fabricate a biosensing platform for lipopolysaccharide (LPS) detection, which successfully detected LPS with very high sensitivity. This indicates the promising application of these biosensors for early detection of endotoxins. Furthermore, the amazing properties of these np-AuNPs make them suitable candidates for future studies in various applied fields, including drug loading and delivery, catalysis, and energy.

## Figures and Tables

**Table 1:** Sizes of np-AuNPs according to composition of gold and silver in alloy nanoparticles.

np-AuNPs According to Molar Au:Ag (Au% in Alloy Nanoparticles)	Average Size of np-AuNPs (nm ± SD)
7:3 (70% Au)	89.18 ± 13.31 (pores were not clearly visible)
np-AuNPs(3:2) (60% Au)	121.49 ± 9.22
np-AuNPs(1:1) (50% Au)	131.01 ± 11.14
np-AuNPs(2:3) (40% Au)	155.90 ± 15.27
np-AuNPs(3:7) (30% Au)	182.38 ± 13.86
np-AuNPs(1:4) (20% Au)	308.81 ± 25.22
np-AuNPs(3:17) (15% Au)	525.39 ± 71.86
1:9 (10% Au)	Ligaments not connected properly to form np-AuNPs

**Table 2:** EDS data showing gold–silver composition in np-AuNPs with dealloying time.

Time	Elment	Atomic %	Atomic % Error	Weight %	Weight % Error
**(A) 4 h**	Ag	19.1	1.7	11.5	1.0
	Au	80.9	0.8	88.5	0.9
**(B) 6 h**	Ag	14.5	2.8	8.5	1.6
	Au	85.5	1.3	91.5	1.4
**(C) 8 h**	Ag	10.2	1.6	5.9	0.9
	Au	89.8	0.9	94.1	1.0
**(D) 10 h**	Ag	7.6	1.9	4.3	1.1
	Au	92.4	1.7	95.7	1.8

**Table 3:** Peak potential and peak current associated with the CV curves in Figure 15.

	Anodic Peak Potential (*E_p,a_*) (V)	Cathodic Peak Potential (*E_p,__c_*) (V)	Anodic Peak Current (*I_p,a_*) (mA)	Cathodic Peak Current (*I_p,__c_*) (mA)	*∆E = E_p,a_* − *E_p,c_*	$\left|\frac{I_{p,a}}{I_{p,c}}\right|$
Bare ITO	0.5422	−0.1742	0.3029	−0.0337	0.7164	8.9908
np-AuNPs (1:1)	0.3679	0.0209	0.6444	−0.5229	0.3470	1.2322
np-AuNPs (2:3)	0.3651	0.0321	0.6616	−0.5518	0.3294	1.1990
np-AuNPs (3:7)	0.2944	0.1137	0.9614	−0.9329	0.1807	1.0305
np-AuNPs (1:4)	0.3167	0.0849	0.9200	−0.7758	0.2319	1.1859

**Table 4:** Charge-transfer resistance (R_ct_) for different analytes immobilized on modified electrode surface.

Analytes on the Electrode Surface	Charge-Transfer Resistance (*R_ct_*) (Ω)
Lipid-A LPS Antibody	176.0
BSA and Antibody	177.5
Con-A Only	208.2
1 ng/L LPS	263.9
5 ng/L LPS	332.6
10 ng/L LPS	420.4
50 ng/L LPS	1112.0
100 ng/L LPS	1574.0
500 ng/L LPS	2261.0
1000 ng/L LPS	3024.3

## Data Availability

The original contributions presented in this study are included in the article. Further inquiries can be directed to the corresponding author.

## Abbreviations

The following abbreviations are used in this manuscript:

APTES	(3-Aminopropyl)triethoxysilane
CPE	Constant phase element
CV	Cyclic voltammetry
DLS	Dynamic light scattering
EDC	N-(3-dimethylaminopropyl)-N′-ethylcarbodiimide
EDS	Energy-dispersive X-ray spectroscopy
EIS	Electrochemical impedance spectroscopy
EU	Endotoxin unit
FCC	Face-centered cubic
GNB	Gram-negative bacteria
HAADF	High-angle annular dark field
ICH	International Council for Harmonisation of Technical Requirements for Pharmaceuticals for Human Use
ITO	Indium tin oxide
LOD	Limit of detection
LOQ	Limit of quantification
LPS	Lipopolysaccharide
LSPR	Localized surface plasmon resonance
NHS	N-hydroxysuccinimide
NIR	Near infrared
np-Au	Nanoporous gold
np-AuNPs	Nanoporous gold nanoparticles
PEG	Polyethylene glycol
PVP	Polyvinylpyrrolidone
SEM	Scanning electron microscopy
STEM	Scanning transmission electron microscopy
TEM	Transmission electron microscopy
